# Targeting dopamine transporter to ameliorate cognitive deficits in Alzheimer's disease

**DOI:** 10.3389/fncel.2023.1292858

**Published:** 2023-11-13

**Authors:** Ammara Shaikh, Fairus Ahmad, Seong Lin Teoh, Jaya Kumar, Mohamad Fairuz Yahaya

**Affiliations:** ^1^Department of Anatomy, Faculty of Medicine, Universiti Kebangsaan Malaysia, Cheras, Kuala Lumpur, Malaysia; ^2^Department of Physiology, Faculty of Medicine, Universiti Kebangsaan Malaysia, Cheras, Kuala Lumpur, Malaysia

**Keywords:** dopamine transporter, dopamine, Alzheimer's disease, mesocorticolimbic pathway, cognition

## Abstract

Alzheimer's disease (AD) is characterized by the pathologic deposition of amyloid and neurofibrillary tangles in the brain, leading to neuronal damage and defective synapses. These changes manifest as abnormalities in cognition and behavior. The functional deficits are also attributed to abnormalities in multiple neurotransmitter systems contributing to neuronal dysfunction. One such important system is the dopaminergic system. It plays a crucial role in modulating movement, cognition, and behavior while connecting various brain areas and influencing other neurotransmitter systems, making it relevant in neurodegenerative disorders like AD and Parkinson's disease (PD). Considering its significance, the dopaminergic system has emerged as a promising target for alleviating movement and cognitive deficits in PD and AD, respectively. Extensive research has been conducted on dopaminergic neurons, receptors, and dopamine levels as critical factors in cognition and memory in AD. However, the exact nature of movement abnormalities and other features of extrapyramidal symptoms are not fully understood yet in AD. Recently, a previously overlooked element of the dopaminergic system, the dopamine transporter, has shown significant promise as a more effective target for enhancing cognition while addressing dopaminergic system dysfunction in AD.

## 1. Introduction

Alzheimer's disease (AD) is a progressive neurodegenerative disorder characterized by cognitive decline and behavioral abnormalities accompanied by impairments in various personality domains. AD has an intricate pathophysiology that is believed to be initiated by oxidative stress, leading to the deposition of amyloid-β (Aβ), tau hyperphosphorylation, neuroinflammation, and neurodegeneration (Tarkowski, 2003; Manczak et al., 2011; Leuner et al., 2012). The neuropathology in AD involves abnormal neuronal circuits (Busche et al., 2019) resulting from aberrant synaptic morphology and dysfunctional neurotransmitter systems (NTS) (Hsieh et al., 2006; Meyer-Luehmann et al., 2008; Kandimalla and Reddy, 2017). The synaptic dysfunction starts with impairment of long-term potentiation and eventually leads to synaptic depression (Selkoe, 2002; Palop and Mucke, 2010). Several NTS, such as acetylcholine, catecholamines (such as dopamine and norepinephrine), indoleamines (such as serotonin), and glutamate, have been associated with cognition, and their abnormal functioning is observed in AD.

The dopaminergic system, being a crucial NTS, exhibits decreased levels of dopamine receptors (Pan et al., 2019), dopamine neurotransmitter, dopaminergic neuronal count, and connectivity in the ventral tegmental area (VTA)—hippocampus—nucleus accumbens (NAc) loop (Nobili et al., 2017; Cordella et al., 2018; Sala et al., 2021) in AD brains. While the administration of dopamine agonists has been found effective in restoring cortical plasticity in AD patients (Koch et al., 2014), it improves only frontal-lobe-related cognition without significantly impacting global cognition (Koch et al., 2020). Moreover, a newly studied component of the dopaminergic system, the dopamine transporter (DAT), has shown promise in increasing dopamine levels and attenuating disease progression when blocked. Based on these findings, we present evidence suggesting that targeting the DAT could be a potential strategy for alleviating cognitive dysfunction in mild to moderate AD.

## 2. Dopaminergic system—Role of dopamine transporter

The dopaminergic system primarily consists of dopaminergic neurons, receptors, the neurotransmitter dopamine, and dopamine transporter. The neurotransmitter released by dopaminergic neurons exerts excitatory and inhibitory effects by acting on presynaptic and postsynaptic receptors, known as dopamine receptors (Juárez Olguín et al., 2016). There are five dopamine receptors, namely D1, D2, D3, D4, and D5, which can be categorized into two groups: D1-like receptors (D1 and D5) coupled to G-stimulatory sites, and D2-like receptors (D2, D3, and D4) coupled to G-inhibitory sites (Bhatia et al., 2023). D1 receptors are the most abundant in the central nervous system, followed by D2, D3, D5, and D4 subtypes (Bhatia et al., 2023). These dopamine receptors are distributed in various brain regions with the possibility of co-existence of different dopamine receptors within the same neuron (Jaber et al., 1996; Perreault et al., 2011).

The D3, D4, and D5 receptors are primarily associated with cognition, while D1 and D2 receptors are linked to learning and memory (Gross and Drescher, 2012; Carr et al., 2017; Mishra et al., 2018). The final component of the dopaminergic system is the DAT, a transmembrane protein located in the presynaptic terminal of dopaminergic neurons responsible for dopamine reuptake. It plays an essential role in regulating synaptic dopamine levels, making it the key regulator of dopaminergic neuron connectivity (Miller et al., 2021). Multiple modulators, including D2 and D3 receptors, influence the dopamine transporter's function. Activation of D2 receptors increases dopamine transporter activity and dopaminergic reuptake (Ramamoorthy et al., 2011), whereas modulation by D3 receptors varies in a biphasic manner, with short-term activation increasing DAT surface expression and prolonged activation leading to inhibition (Zapata et al., 2007).

## 3. The anatomy and physiology of dopamine transporter

The DAT is a transmembrane protein belonging to the family of Na^+^/Cl- -dependent neurotransmitter transporters and comprises 12 helices. These transmembrane helices (TMH) are interconnected through intracellular and extracellular loops (Bu et al., 2021). As a membrane-spanning protein belonging to solute carrier 6 transport family, the DAT undergoes conformational change in response to ligand binding (Shan et al., 2011). This change in its conformation is necessary for the translocation of dopamine into the neuron. Hence, blocking of this conformation results in dopamine efflux outside the neuron, as happens in response to cocaine binding (Huang et al., 2009). There are two main sites for ligand binding, a central or primary substrate binding site (S1) and a vestibular or secondary substrate binding site (S2), the latter of which greatly influences former's function (Shi et al., 2008; Shan et al., 2011). The antagonist attachment to the S1 competitively inhibits ligand binding, whereas the antagonist occupancy of S2 allosterically prevents ligand transport through DAT. Therefore, S1 and S2 along with other allosteric sites are the targets for the DAT modulators (Nepal et al., 2023).

Physiologically, the influx of dopamine requires its attachment with S1 in the presence of two Na+ and one Cl^−^ ion (Shan et al., 2011). The S1 binding of dopamine induces inward conformation of DAT that results in influx of released dopamine from the synaptic cleft back into the presynaptic neuron by coupling Na+ out of the cell and dopamine back into the axonal terminal (Shi et al., 2008). This process terminates dopamine neurotransmission and regulates the duration of dopamine's effect on its corresponding receptors. The TMH contain substrate binding sites for dopamine regulation extracellularly, with the larger Amino (N-) and Carboxy (C-) terminals extending to the cytoplasm to modulate the function of the DAT intracellularly (Vaughan and Foster, 2013).

The N-terminus contains residues for phosphorylation (Khoshbouei et al., 2004; German et al., 2015) and ubiquitination (German et al., 2015), while the C-terminus contains interaction domains for various proteins. These proteins include Parkin, which controls the cell surface expression of the DAT (Jiang et al., 2004), and α-synuclein, which regulates intracellular dopamine levels and modulates dopaminergic neuronal apoptosis (Dagra et al., 2021). Additionally, there are binding sites for lipid-raft protein-Flotillin and Ras-like protein Rin, which act as mediators of membrane mobility (Sorkina et al., 2013), and protein kinase C (PKC), which triggers endocytosis of the DAT (Navaroli et al., 2011).

**Figure 1:**
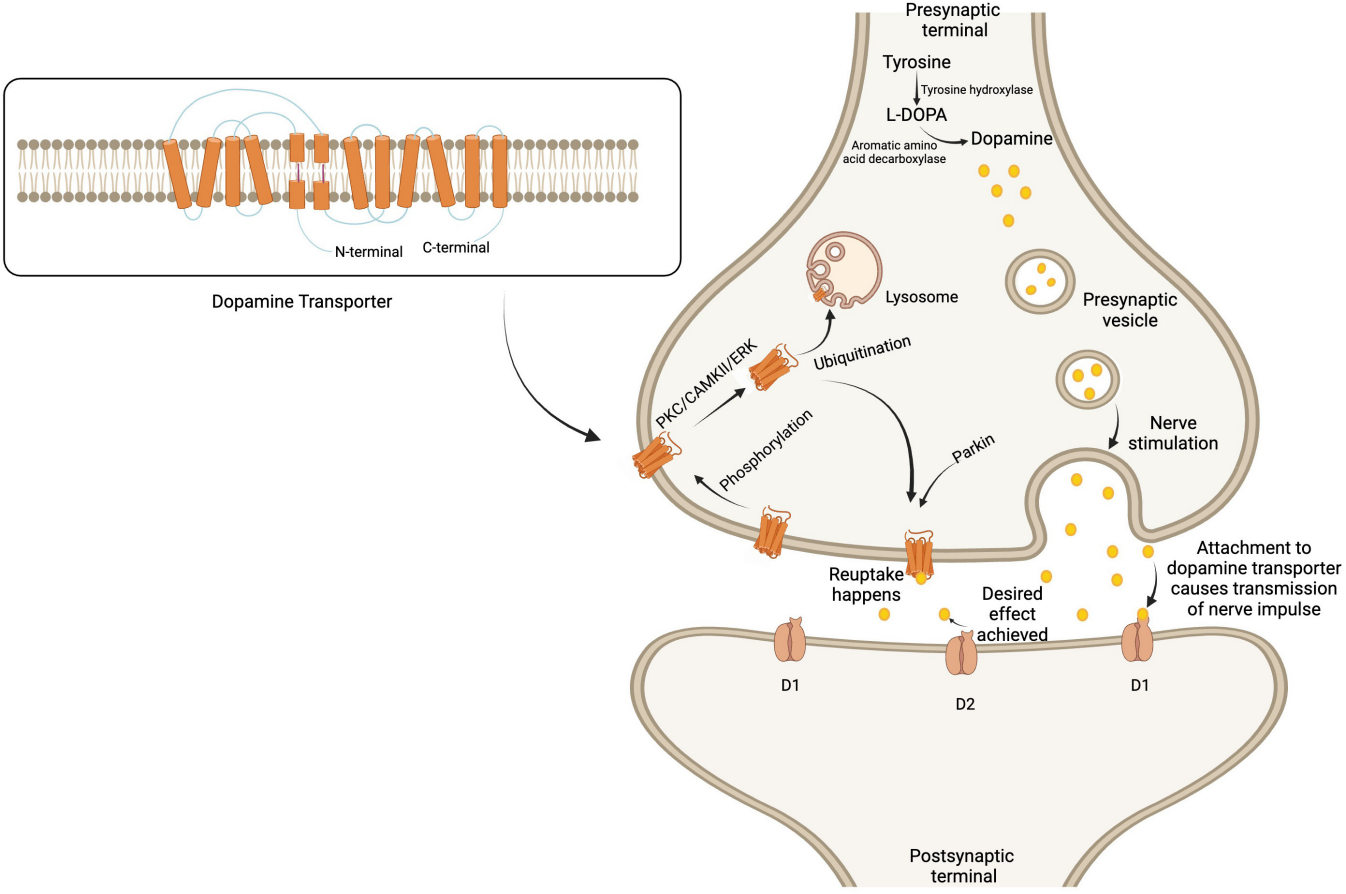
Regulation of dopamine metabolism and dopamine transporter surface expression in the synaptic cleft. Dopamine is formed from tyrosine and stored in the presynaptic vesicles to be released upon nerve stimulation. The secreted dopamine in the synaptic cleft is attached to the post-synaptic dopamine receptors to transmit the nerve impulse. Soon after achieving the desired effect, the dopamine transporter reuptake results in the termination of signals. The expression of the dopamine transporter is regulated by phosphorylation, followed by internalization through any of the three kinase pathways. The transporter then undergoes ubiquitination, which leads to either degradation by lysosomes or recycling back to the membrane. Created with BioRender.com.

Regarding post-translational modification, the DAT protein in dopaminergic pathways is continuously regulated by phosphorylation, followed by internalization, through three main kinase pathways: PKC, calcium-calmodulin dependent kinase II, and extracellular signal-regulated protein kinase (ERK) (Fog et al., 2006; German et al., 2015). After phosphorylation-mediated internalization, the DAT undergoes ubiquitination, a process crucial for protein homeostasis. The N-terminus determines whether the DAT will be recycled back onto the cell surface or completely degraded by the action of lysosomes (Boudanova et al., 2008; German et al., 2015), depending on the demands of the dopaminergic pathway (Figure 1).

On the other hand, dopamine synthesis depends on the activity of tyrosine hydroxylase (TH), which serves as the rate-limiting enzyme for converting the amino acid tyrosine into dopamine (Xiao et al., 2021). The DAT not only facilitates the degradation of dopamine, promoting its reuptake, but may also affect the levels of TH (Salvatore and Pruett, 2012; Salvatore et al., 2016). In this way, TH and the DAT work synchronously to maintain the desired levels of dopamine in the dopaminergic pathways of the brain. Moreover, the dopamine transport DAT is also responsible for modulating membrane potential of the cell and hence the neuronal function in the dopaminergic pathways (Carvelli et al., 2004).

The DAT primarily regulates dopamine in most areas of the dopaminergic pathway. However, in specific regions like the hippocampus, it is mainly metabolized by norepinephrine transporter (NET) (Borgkvist et al., 2012), and in the prefrontal cortex (PFC), it is metabolized by Catechol-O-Methyltransferase (COMT) due to the scarcity of dopamine transporter in these areas (Lammel et al., 2008).

## 4. Mesocorticolimbic circuitry: the interplay of dopamine and the dopamine transporter in cognition

The brain has three main dopaminergic pathways: the nigrostriatal (NS), the mesocorticolimbic (MCL), and the tuberoinfundibular. While the role of dopaminergic modulation in cognition is not fully understood, dopamine in the striatum, midbrain, limbic system, and PFC is believed to be involved in memory and cognition through the NS and MCL pathways (Schott et al., 2006; McNamara et al., 2014; Herrera et al., 2020; Vassilev et al., 2021).

In the NS pathway, there is a dopamine connection between the substantia nigra and the striatum, while in the MCL pathway, the midbrain, limbic system, and PFC are interconnected through dopamine. The MCL circuit itself consists of two pathways: the mesocortical pathway, where dopamine neurons have their cell bodies in the VTA of the midbrain and extend nerve fibers to the PFC, and the mesolimbic pathway, where nerve fibers project to the NAc and other limbic structures (Krashia et al., 2022). Physiologically regulated dopamine innervation and neurotransmission are crucial for the proper functioning of these dopaminergic pathways. Reduction in dopaminergic neurons and/or dopamine neurotransmitter levels can lead to defective connectivity between linking areas.

Similar to NS pathway, the MCL pathway also plays a vital role in cognition, including learning, memory, and decision-making, modulated by dopamine activity in the frontal lobe, limbic system, and midbrain (McNamara et al., 2014; Engelhard et al., 2019; Coddington et al., 2023). Therefore, alterations in dopamine levels may likely contribute to cognitive deficits (Koch et al., 2014; Pan et al., 2019).

Dopamine signals are primarily terminated by either reuptake via transporters or enzymatic degradation by COMT (Caire et al., 2023). Altered levels of these dopamine-signal terminators can also impact cognition, as observed with the DAT inhibitors that improve memory and cognition in neurodegenerative diseases (as described below). Paradoxically, lower dopamine transporter levels have been observed in some psychiatric disorders, such as attention deficit hyperactivity disorder (Kurzina et al., 2020) and depression (Pizzagalli et al., 2019; Dubol et al., 2020). This paucity of dopamine in the absence of higher dopamine transporter in these disorders may be due to high extracellular levels of dopamine that eventually lead to an inability to replenish dopamine in synaptic vesicles, causing a lower amplitude of dopamine release per nerve impulse (Benoit-Marand et al., 2000). Additionally, other factors, such as interaction with α-synuclein in PD, can decrease dopamine transporter activity without affecting its concentration at the plasma membrane, resulting in reduced dopamine reuptake and subsequent lower extracellular dopamine (Swant et al., 2011; Pahrudin Arrozi et al., 2017). Furthermore, dopamine neuronal damage and degeneration may also be associated with decreased levels of the DAT, even without any defect in its activity (Cheng et al., 2010; Fazio et al., 2018).

Overall, both increased and decreased dopamine levels can lead to unwanted symptoms, highlighting the importance of maintaining a continuous check-and-balance of dopamine in synapses for the normal functioning of the dopaminergic pathways.

## 5. Modifications in mesocorticolimbic circuitry and dopamine transporter: implications for AD

Physiological aging leads to changes in the MCL circuitry, characterized by decreased dopamine levels, reduced expression of dopamine receptors, and synaptic dysfunction (Volkow et al., 1996; Kaasinen, 2000; Norrara et al., 2018). The normal aging process is also associated with a decline in the DAT level in certain brain regions, including the hippocampus, PFC, and putamen (Volkow et al., 1996). However, these alterations in the MCL loop are more pronounced in AD, mainly affecting the dopaminergic neuronal count and dopamine receptors' expression, except for D5, which is probably increased in the frontal lobe (Kumar and Patel, 2007).

Subsequently, the MCL loop neuropathology leads to decreased dopamine connectivity and impaired long-term potentiation in AD brain (Koch et al., 2014). Additionally, the damage to pyramidal neurons and synapses in the hippocampus and PFC, due to progressive neurodegeneration caused by amyloid plaque deposition and tau pathology, contribute to impaired cognition and memory (Kemppainen et al., 2003; Ambrée et al., 2009; Guzmán-Ramos et al., 2012).

Although various abnormalities were observed in the MCL pathway, no change in DAT activity was found in AD (Joyce et al., 1997). However, a newer molecular imaging study reported decreased DAT density and activity in both the MCL loop and caudate nucleus in the defective dopaminergic system in the AD brain (Sala et al., 2021). Despite the possibility of already reduced DAT levels, further blocking the DAT improved cognitive deficits, as recently observed in animal models of aging and AD (Xu et al., 2021; Yin et al., 2023). Even though several studies deduced temporary improvement in cognition due to increased synaptic dopamine, newer DAT blockers can repair cognitive deficits by reducing the disease's neuropathology. Although the exact mode of action is yet to be elucidated, the cognitive improvement is thought to be due to the inhibition of α-synuclein and Aβ_1 − 42_ aggregation in the hippocampus and the promotion of lysosomal biogenesis and subsequent degradation of Aβ plaques (Xu et al., 2021; Yin et al., 2023).

Although the MCL pathway and the NS pathway may exhibit distinct roles, the DAT activity and the regulated level of synaptic dopamine are equally crucial for the normal functioning of both. The importance of DAT in MCL has yet to be explored so far, likely due to its negligible presence in some of the areas. However, its reduced levels in the NS loop are linked with cognitive impairment (Li et al., 2020; Fiorenzato et al., 2021). The DAT is found to be closely associated with cognition, as the uninhibited blockade or elimination of the DAT may worsen the disease pathology, as observed in DAT knock-out rodents showing severe cognitive deficits (Leo et al., 2018; Kurzina et al., 2020). Therefore, controlled inhibition of DAT function is crucial to avoid disturbing its physiological effects in the AD brain.

## 6. Effects of dopamine transporter modulators on cognition in AD

The DAT modulators with the potential to enhance cognition and memory can be classified into two main categories. The first category includes substrate-like competitive inhibitors that reduce dopamine reuptake and increase dopamine efflux. The second category comprises atypical or highly-specific DAT inhibitors that prevent dopamine reuptake, increasing dopamine concentration in synapses (Goodwin et al., 2009).

Surprisingly, none of these agents have been studied in neurodegenerative diseases despite their efficacy in improving cognition. Likewise, modafinil (diphenylmethyl-sulfinylacetamide), a prototype of a non-specific DAT inhibitor that also acts on NET and serotonin transporter in the striatum (Madras et al., 2006), has not garnered much interest from researchers in this field. In contrast, Yin et al. (2023) recently introduced a novel class of DAT modulators that exert their effects on DAT and lysosomal activity and, hence, can be termed “DAT-inhibitors-and-lysosomal-activity-promoters- (DILAP)”. These drugs were tested on AD mice and effectively improved memory and cognitive deficits. Moreover, DILAP were also found to reduce intracerebral Aβ burden by promoting lysosomal synthesis and phagocytosis (Yin et al., 2023). Two examples of DILAP are the lysosome-enhancing compound LH2-051 and clomipramine hydrochloride (HCl), also known as Anafranil or S2541. LH2-051 inhibits the DAT, and its binding leads to translocation of the DAT from the plasma membrane to the lysosomal membrane via intracellular vesicles. The localization of DAT onto lysosome decreases the availability of the phosphorylating proteins and, therefore, promotes dephosphorylation of transcription factor EB (TFEB). The dephosphorylated TFEB then undergoes nuclear translocation, which enhances the expression of lysosomal and autophagic genes, promoting lysosomal acidification and biogenesis (Yin et al., 2023). This increase in active lysosomes ultimately results in Aβ clearance and improved learning, memory, and cognition (Yin et al., 2023). The mentioned outcomes of dopamine transport inhibition and lysosomal activation are comparable with the effects of Clomipramine HCl that blocks the activity of the DAT along with the serotonin transporter and NET (Gillman, 2007; Yin et al., 2023). In this way, it may also be effective in attenuating AD neuropathology.

## 7. Implications of recent studies on alternative dopamine transporter inhibitors

The modulators acting as substrate-like agents for the DAT include amphetamine and methamphetamine, while DAT-specific agents comprise modafinil derivatives better known as (synthetic) modafinil analogs. Although both amphetamine and methamphetamine are non-specific, having more affinity NET receptors, they are potent inhibitors of DAT (Han and Gu, 2006; Docherty and Alsufyani, 2021). By acting as substrate-like competitive inhibitors, these agents can decrease dopamine reuptake and increase dopamine efflux (Goodwin et al., 2009) in brain regions such as the medial PFC (mPFC), dentate gyrus (DG) (Fog et al., 2006; Shyu et al., 2021), and NAc (Hedges et al., 2018). On the other hand, DAT-specific drugs selectively target DAT-mediated reuptake in the mPFC (Sagheddu et al., 2020; Kouhnavardi et al., 2022), NAc (Kouhnavardi et al., 2022), and hippocampus (Kristofova et al., 2018).

**Figure 2:**
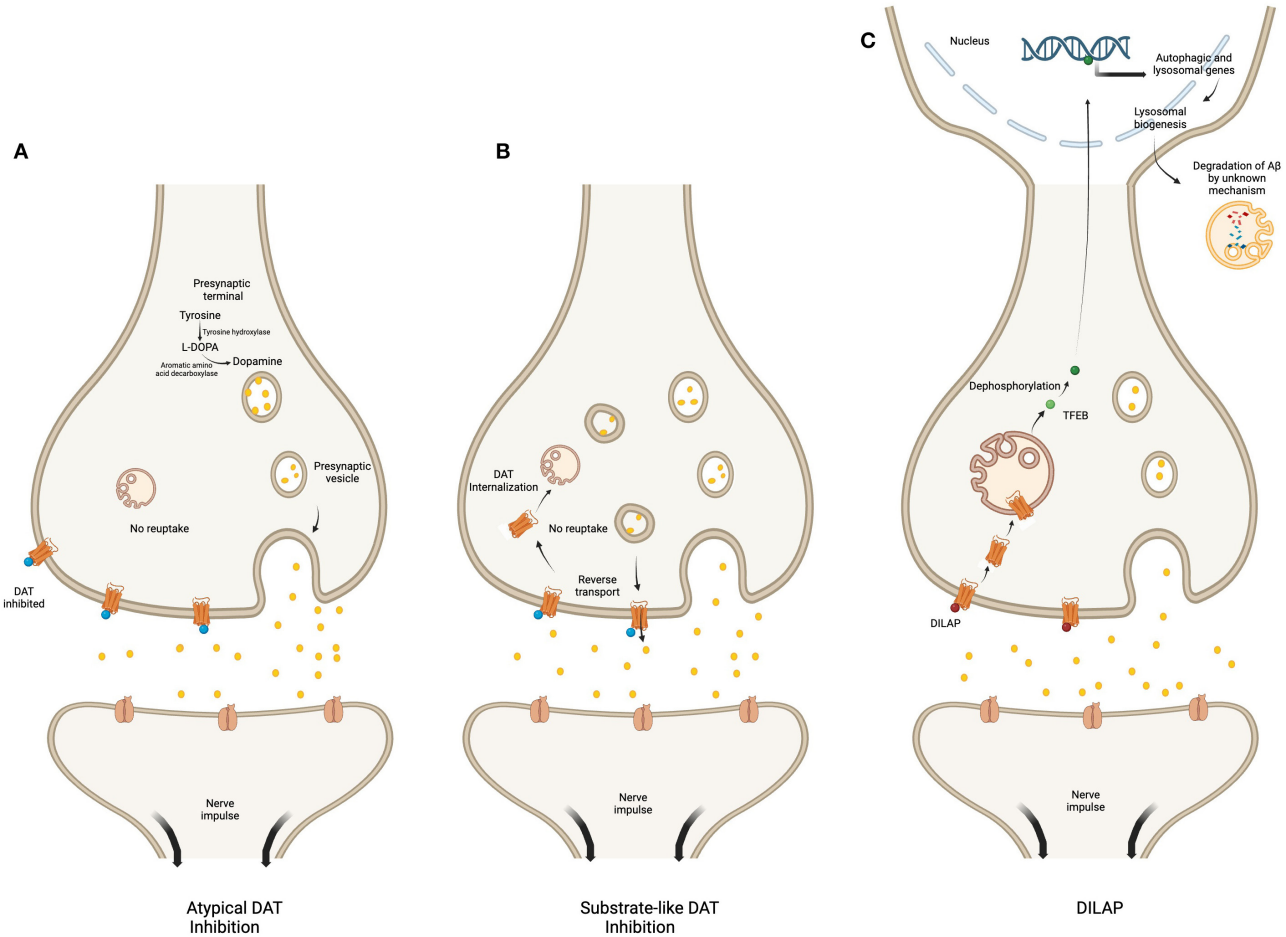
Regulatory mechanism of various DAT inhibitors. The figure compares the mechanism of action of atypical DAT inhibitor **(A)**, substrate-like DAT inhibitor **(B)**, and DILAP **(C)**. The atypical inhibitors bind DAT to block dopamine reuptake **(A)**. The substrate-like inhibitors block dopamine influx while stimulating the DAT-mediated reverse transport of DAT into the synaptic cleft; they also decrease DAT availability by promoting its internalization **(B)**. The DILAP inhibits DAT to increase synaptic dopamine, and their binding cause DAT translocation to the lysosomal membrane, which results in nuclear translocation of the dephosphorylated TFEB to increase expression of autophagic and lysosomal genes, ultimately leading to Aβ-plaques degradation by an unknown mechanism **(C)**. Created with BioRender.com.

The mechanism of action of DAT inhibition is different among the three classes of drugs, i.e., atypical inhibitors, substrate-like competitive inhibitors and DILAP. The atypical DAT inhibitors increase the synaptic dopamine level by inhibiting DAT function (Loland et al., 2012). Whereas, the substrate-like inhibitors, like amphetamine increase synaptic dopamine levels by inhibiting DAT uptake, promoting DAT mediated reverse-transport of dopamine and facilitating exocytic dopamine release (Calipari and Ferris, 2013; Daberkow et al., 2013). Additionally, they may also stimulate internalization of the plasma membrane DAT, thereby further decreasing its availability and function (Wheeler et al., 2015). In comparison, the DILAP inhibit the DAT mediated dopamine reuptake, while promoting its translocation from the plasma membrane to the lysosomal membrane. This translocation increases expression of lysosomal and autophagic genes which promotes degradation of Aβ-plaques (Yin et al., 2023) (Figure 2).

Due to their high DAT specificity, synthetic modafinil analogs like R-modafinil, S-CE-123 (S-5-((benzhydrylsulfinyl)methyl)thiazole), S,S-CE158 (5-(((S)-((S)-(3-bromophenyl)(phenyl)methyl)sulfinyl)methyl)thiazole), and S-MK-26 ((S)-5-(((B(3-chlorophenyl)methyl)sulphinyl)methyl)thiazole) do not exert any effect on the reward pathway, making them less likely to cause addiction, abuse or withdrawal symptoms compared to the parent drug and other non-specific counterparts (Kristofova et al., 2018; Sagheddu et al., 2020; Hazani et al., 2022; Kouhnavardi et al., 2022). These modafinil analogs also have the potential to improve synaptic transmission and plasticity in the hippocampus (Kouhnavardi et al., 2022).

Considering that studies evaluating the effects of DAT inhibition showed improved cognition attributed to amyloid and α-synuclein in the MCL pathway, especially in the hippocampus and cortex (Xu et al., 2021; Yin et al., 2023), it is likely that other DAT inhibitors may exert a similar effect in the AD brain. As atypical DAT inhibitors also improve synaptic plasticity while having a minimal tendency for addiction, they could be prime candidates to be tested on AD rodent models for cognitive improvement.

## 8. How dopamine transporter can be a better target?

Previous studies on AD brains have shown normal and reduced levels of DAT (Joyce et al., 1997; Sala et al., 2021) and no change in DAT sites and TH levels in the VTA (Murray et al., 1995). Currently, there is no drug approved by the FDA for the treatment of the dopaminergic system in AD (Chopade et al., 2023), as both the L-Dopa and dopamine agonists failed to demonstrate compelling results to improve cognition in the elderly (Lebedev et al., 2020) and AD subjects (Koch et al., 2020), respectively. In comparison, the newer studies targeting the DAT in AD animal models have shown promise (Xu et al., 2021; Yin et al., 2023).

Since dopamine is involved in the NS pathway, which affects mood and motivation, the effects of DAT inhibitors should also be considered in that loop. A study by Udo et al. found an inverse correlation between the level of DAT in the caudate nucleus and the severity of apathy in AD (Udo et al., 2020). However, it is worth noting that the study mentioned the degeneration of dopaminergic neurons in the NS pathway as the cause of decreased DAT levels in presynaptic terminals. Therefore, apathy is likely due to the loss of these striatal dopaminergic neurons (Udo et al., 2020). In line with this notion, a newer study demonstrated the potential of specific DAT inhibitors to improve motivational impairments in rats (Kouhnavardi et al., 2022). Thus, DAT inhibitors may not worsen apathy, as the symptom is primarily a result of dopamine neuronal degeneration in the NS pathway.

Moreover, the development of novel heterocyclic compounds, such as CE-125 (4-((benzhydrylsulfinyl)methyl)-2-cyclopropylthiazole)) and CE-111 (4-(benzhydrylsulfinylmethyl)-2-methyl-thiazole), acting as DAT-specific inhibitors along with dopamine receptor modulators, have shown potential as better alternatives to modafinil-derived analogs by having multiple targets in the dysfunctional dopaminergic system (Saroja et al., 2016; Hussein et al., 2017). These atypical DAT inhibitors may effectively improve cognition in cases of defective dopaminergic pathways. Furthermore, the effects of DAT modulation for cognitive enhancement can be evaluated by targeting specific binding sites, which is now feasible after the identification of novel allosteric sites on the DAT protein (Cheng et al., 2017; Aggarwal et al., 2019).

## 9. Discussion and future directions

Studies found that the MCL pathway is affected more than the NS pathway in AD (Koeppe et al., 2008; Colloby et al., 2012; Sala et al., 2021). Therefore, targeting the abnormal MCL pathway can alleviate most dopamine-related cognitive deficits. For this purpose, DAT inhibitors can be considered as potential targets to improve cognition by reducing the dopamine reuptake. However, while inhibiting DAT, the precise regulation of dopamine in the MCL loop must be ensured, especially in the VTA-hippocampal circuit, to avoid dopamine disbalance and resulting adverse effects. If, for instance, DAT is excessively blocked, then the resultant hyperdopaminergic state can lead to dopamine dysregulation syndrome, promoting undesirable responses like gambling and drug addiction. Conversely, a hypodopaminergic state can lead to anxiety and apathy (Calabresi et al., 2013). Care must also be considered when generalizing DAT inhibition to the whole brain as certain chemical can downregulate DAT as well as TH gene expression (Mohamad Najib et al., 2023). However, considering that the dopamine is metabolized by COMT and NET in the PFC and hippocampus, respectively (Lammel et al., 2008; Borgkvist et al., 2012), the regulated substrate modification of the DAT would not severely alter the dopamine levels in these areas.

Presently, no drug is approved to treat the dopaminergic system-related cognitive deficits in AD. However, recent studies on DAT inhibitors demonstrate its potential to be an efficacious target, with the newly discovered DILAP possessing the ability to restore cognitive deficits by ameliorating AD neuropathology. The DILAP binds to DAT causing translocation of the latter to the lysosomal membrane in order to increase the lysosomal biogenesis and subsequent dissolution of amyloid plaque by a mechanism not understood yet. The exact mechanism of action of DILAP to improve cognition is yet to be elucidate, however, it is suggested to be linked with the attenuation of amyloid burden. Moreover, a study on an AD mouse model showed promising effects of Nilotinib, a tyrosine kinase inhibitor that reduces Aβ levels, prevents structural damage and degeneration of dopaminergic neurons in the VTA, and restores VTA-hippocampal loop function, thereby reducing dopamine-related cognitive impairments (Barbera et al., 2021). This points toward the possibility of developing useful nootropics in combination with DAT inhibitors to improve MCL pathway functioning, ameliorate cognitive impairment, and mitigate AD neuropathology simultaneously. Furthermore, atypical DAT inhibitors can be tested with agents such as acetylcholinesterase inhibitors and N-methyl-D-aspartate receptor antagonists. Positive results from such combinations could help improve cognitive impairment and decrease the pathologic burden of the disease in the brain.

## Author contributions

AS: Conceptualization, Methodology, Writing – original draft. MY: Conceptualization, Formal analysis, Funding acquisition, Project administration, Supervision, Writing – review & editing. FA: Writing – review & editing. JK: Writing – review & editing. ST: Writing – review & editing.

## References

[B1] Aggarwal S. Liu X. Rice C. Menell P. Clark P. J. Paparoidamis N.. (2019). Identification of a novel allosteric modulator of the human dopamine transporter. ACS Chem. Neurosci. 10, 3718–3730. 10.1021/acschemneuro.9b00262 31184115 PMC6703927

[B2] Ambrée O. Richter H. Sachser N. Lewejohann L. Dere E. Souza Silva M. A.. (2009). Levodopa ameliorates learning and memory deficits in a murine model of Alzheimer's disease. Neurobiol. Aging 30, 1192–1204. 10.1016/j.neurobiolaging.2007.11.010 18079024

[B3] Barbera L. Vedele F. Nobili A. Krashia P. Spoleti E. Latagliata E. C.. (2021). Nilotinib restores memory function by preventing dopaminergic neuron degeneration in a mouse model of Alzheimer's disease. Progr. Neurobiol. 202, 102031. 10.1016/j.pneurobio.2021.102031 33684513

[B4] Benoit-Marand M. Jaber M. Gonon F. (2000). Release and elimination of dopamine in vivo in mice lacking the dopamine transporter: functional consequences: dopamine release in mice lacking DA transporter. Eur. J. Neurosci. 12, 2985–2992. 10.1046/j.1460-9568.2000.00155.x 10971639

[B5] Bhatia A. Lenchner J. R. Saadabadi A. (2023). “Biochemistry, dopamine receptors,” in StatPearls (StatPearls Publishing). 30855830

[B6] Borgkvist A. Malmlöf T. Feltmann K. Lindskog M. Schilström B. (2012). Dopamine in the hippocampus is cleared by the norepinephrine transporter. Int. J. Neuropsychopharmacol. 15, 531–540. 10.1017/S1461145711000812 21669025

[B7] Boudanova E. Navaroli D. M. Stevens Z. Melikian H. E. (2008). Dopamine transporter endocytic determinants: carboxy terminal residues critical for basal and PKC-stimulated internalization. Molec. Cell. Neurosci. 39, 211–217. 10.1016/j.mcn.2008.06.011 18638559 PMC2585501

[B8] Bu M. Farrer M. J. Khoshbouei H. (2021). Dynamic control of the dopamine transporter in neurotransmission and homeostasis. NPJ Parkinson's Dis. 7, 22. 10.1038/s41531-021-00161-2 33674612 PMC7935902

[B9] Busche M. A. Wegmann S. Dujardin S. Commins C. Schiantarelli J. Klickstein N.. (2019). Tau impairs neural circuits, dominating amyloid-β effects, in Alzheimer models in vivo. Nat. Neurosci. 22, 57–64. 10.1038/s41593-018-0289-8 30559471 PMC6560629

[B10] Caire M. J. Reddy V. Varacallo M. (2023). “Physiology, synapse,” in StatPearls (StatPearls Publishing). 30252303

[B11] Calabresi P. Castrioto A. Filippo M. Picconi B. (2013). New experimental and clinical links between the hippocampus and the dopaminergic system in Parkinson's disease. Lancet Neurol. 12, 811–821. 10.1016/S1474-4422(13)70118-2 23867199

[B12] Calipari E. S. Ferris M. J. (2013). Amphetamine mechanisms and actions at the dopamine terminal revisited. J. Neurosci. 33, 8923–8925. 10.1523/JNEUROSCI.1033-13.2013 23699503 PMC3753078

[B13] Carr G. V. Maltese F. Sibley D. R. Weinberger D. R. Papaleo F. (2017). The dopamine D5 receptor is involved in working memory. Front. Pharmacol. 8, 666. 10.3389/fphar.2017.00666 29056909 PMC5635435

[B14] Carvelli L. McDonald P. W. Blakely R. D. DeFelice L. J. (2004). Dopamine transporters depolarize neurons by a channel mechanism. Proc. Nat. Acad. Sci. USA. 101, 16046–16051. 10.1073/pnas.0403299101 15520385 PMC528740

[B15] Cheng H.-C. Ulane C. M. Burke R. E. (2010). Clinical progression in parkinson's disease and the neurobiology of axons. Ann. Neurol. 67, 715–725. 10.1002/ana.21995 20517933 PMC2918373

[B16] Cheng M. H. Garcia-Olivares J. Wasserman S. DiPietro J. Bahar I. (2017). Allosteric modulation of human dopamine transporter activity under conditions promoting its dimerization. J. Biol. Chem. 292, 12471–12482. 10.1074/jbc.M116.763565 28584050 PMC5535022

[B17] Chopade P. Chopade N. Zhao Z. Mitragotri S. Liao R. Chandran Suja V. (2023). Alzheimer's and Parkinson's disease therapies in the clinic. Bioeng. Transl. Med. 8, e10367. 10.1002/btm2.10367 36684083 PMC9842041

[B18] Coddington L. T. Lindo S. E. Dudman J. T. (2023). Mesolimbic dopamine adapts the rate of learning from action. Nature 614, 294–302. 10.1038/s41586-022-05614-z 36653450 PMC9908546

[B19] Colloby S. J. McParland S. O'Brien J. T. Attems J. (2012). Neuropathological correlates of dopaminergic imaging in Alzheimer's disease and Lewy body dementias. Brain 135, 2798–2808. 10.1093/brain/aws211 22961551

[B20] Cordella A. Krashia P. Nobili A. Pignataro A. La Barbera L. Viscomi M. T.. (2018). Dopamine loss alters the hippocampus-nucleus accumbens synaptic transmission in the Tg2576 mouse model of Alzheimer's disease. Neurobiol. Dis. 116, 142–154. 10.1016/j.nbd.2018.05.006 29778899

[B21] Daberkow D. P. Brown H. D. Bunner K. D. Kraniotis S. A. Doellman M. A. Ragozzino M. E.. (2013). Amphetamine paradoxically augments exocytotic dopamine release and phasic dopamine signals. J. Neurosci. 33, 452–463. 10.1523/JNEUROSCI.2136-12.2013 23303926 PMC3711765

[B22] Dagra A. Miller D. R. Lin M. Gopinath A. Shaerzadeh F. Harris S.. (2021). α-Synuclein-induced dysregulation of neuronal activity contributes to murine dopamine neuron vulnerability. NPJ Parkinson's Dis. 7, 76. 10.1038/s41531-021-00210-w 34408150 PMC8373893

[B23] Docherty J. R. Alsufyani H. A. (2021). Pharmacology of drugs used as stimulants. J. Clin. Pharmacol. 61, S53–S69. 10.1002/jcph.1918 34396557

[B24] Dubol M. Trichard C. Leroy C. Granger B. Tzavara E. T. Martinot J. L.. (2020). Lower midbrain dopamine transporter availability in depressed patients: Report from high-resolution PET imaging. J. Affect. Disor. 262, 273–277. 10.1016/j.jad.2019.10.041 31732277

[B25] Engelhard B. Finkelstein J. Cox J. Fleming W. Jang H. J. Ornelas S.. (2019). Specialized coding of sensory, motor and cognitive variables in VTA dopamine neurons. Nature 570, 509–513. 10.1038/s41586-019-1261-9 31142844 PMC7147811

[B26] Fazio P. Svenningsson P. Cselényi Z. Halldin C. Farde L. Varrone A.. (2018). Nigrostriatal dopamine transporter availability in early parkinson's disease: nigro-striatal degeneration in early phases of PD. Movement Disor. 33, 592–599. 10.1002/mds.27316 29436751

[B27] Fiorenzato E. Antonini A. Bisiacchi P. Weis L. Biundo R. (2021). Asymmetric dopamine transporter loss affects cognitive and motor progression in Parkinson's disease. Movem. Disor. 36, 2303–2313. 10.1002/mds.28682 34124799 PMC8596815

[B28] Fog J. U. Khoshbouei H. Holy M. Owens W. A. Vaegter C. B. Sen N.. (2006). Calmodulin kinase II interacts with the dopamine transporter c terminus to regulate amphetamine-induced reverse transport. Neuron 51, 417–429. 10.1016/j.neuron.2006.06.028 16908408

[B29] German C. L. Baladi M. G. McFadden L. M. Hanson G. R. Fleckenstein A. E. (2015). Regulation of the dopamine and vesicular monoamine transporters: pharmacological targets and implications for disease. Pharmacol. Rev. 67, 1005–1024. 10.1124/pr.114.010397 26408528 PMC4630566

[B30] Gillman P. K. (2007). Tricyclic antidepressant pharmacology and therapeutic drug interactions updated. Br. J. Pharmacol. 151, 737–748. 10.1038/sj.bjp.0707253 17471183 PMC2014120

[B31] Goodwin J. S. Larson G. A. Swant J. Sen N. Javitch J. A. Zahniser N. R.. (2009). Amphetamine and methamphetamine differentially affect dopamine transporters in vitro and in vivo. J. Biol. Chem. 284, 2978–2989. 10.1074/jbc.M805298200 19047053 PMC2631950

[B32] Gross G. Drescher K. (2012). “The role of dopamine D3 receptors in antipsychotic activity and cognitive functions,” in Novel Antischizophrenia Treatments, eds. M.A. Geyer and G. Gross (Berlin Heidelberg: Springer), 167–210. 10.1007/978-3-642-25758-2_7 23027416

[B33] Guzmán-Ramos K. Moreno-Castilla P. Castro-Cruz M. McGaugh J. L. Martínez-Coria H. LaFerla F. M.. (2012). Restoration of dopamine release deficits during object recognition memory acquisition attenuates cognitive impairment in a triple transgenic mice model of Alzheimer's disease. Lear. Memory 19, 453–460. 10.1101/lm.026070.112 22984283

[B34] Han D. D. Gu H. H. (2006). Comparison of the monoamine transporters from human and mouse in their sensitivities to psychostimulant drugs. BMC Pharmacol. 6, 1–7. 10.1186/1471-2210-6-6 16515684 PMC1448202

[B35] Hazani H. M. Naina Mohamed I. Muzaimi M. Mohamed W. Yahaya M. F. Teoh S. L.. (2022). Goofballing of opioid and methamphetamine: The science behind the deadly cocktail. Front. Pharmacol. 13, 859563. 10.3389/fphar.2022.859563 35462918 PMC9021401

[B36] Hedges D. M. Obray J. D. Yorgason J. T. Jang E. Y. Weerasekara V. K. Uys J. D.. (2018). Methamphetamine induces dopamine release in the nucleus accumbens through a sigma receptor-mediated pathway. Neuropsychopharmacology 43, 6. 10.1038/npp.2017.291 29185481 PMC5916361

[B37] Herrera M. L. Deza-Ponzio R. Ghersi M. S. Villarmois E. A. Virgolini M. B. Pérez M. F.. (2020). Early cognitive impairment behind nigrostriatal circuit neurotoxicity: are astrocytes involved?” *ASN NEURO* 12, 1759091420925977. 10.1177/1759091420925977 PMC7263115 32466659

[B38] Hsieh H. Boehm J. Sato C. Iwatsubo T. Tomita T. Sisodia S.. (2006). AMPA-R removal underlies Aβ-induced synaptic depression and dendritic spine loss. Neuron 52, 831–843. 10.1016/j.neuron.2006.10.035 17145504 PMC1850952

[B39] Huang X. Gu H. H. Zhan C. G. (2009). Mechanism for cocaine blocking the transport of dopamine: insights from molecular modeling and dynamics simulations. J. Phys. Chem. B 113, 15057–15066. 10.1021/jp900963n 19831380 PMC2774931

[B40] Hussein A. M. Aher Y. D. Kalaba P. Aher N. Y. Dragačević V. Radoman B.. (2017). A novel heterocyclic compound improves working memory in the radial arm maze and modulates the dopamine receptor D1r in frontal cortex of the sprague-dawley rat. Behav. Brain Res. 332, 308–315. 10.1016/j.bbr.2017.06.023 28629964

[B41] Jaber M. Robinson S. W. Missale C. Caron M. G. (1996). Dopamine receptors and brain function. Neuropharmacology 35, 1503–1519. 10.1016/S0028-3908(96)00100-1 9025098

[B42] Jiang H. Jiang Q. Feng J. (2004). Parkin increases dopamine uptake by enhancing the cell surface expression of dopamine transporter. J. Biol. Chem. 279, 54380–54386. 10.1074/jbc.M409282200 15492001

[B43] Joyce J. N. Smutzer G. Whitty C. J. Myers A. Bannon M. J. (1997). Differential modification of dopamine transporter and tyrosine hydroxylase mRNAs in midbrain of subjects with Parkinson's, Alzheimer's with parkinsonism, and Alzheimer's disease. Movement Disor. 12, 885–897. 10.1002/mds.870120609 9399211

[B44] Juárez Olguín H. Calderón Guzmán D. Hernández García E. Barragán Mejía G. (2016). The role of dopamine and its dysfunction as a consequence of oxidative stress. Oxidat. Med. Cell. Long. 9730467. 10.1155/2016/9730467 26770661 PMC4684895

[B45] Kaasinen V. (2000). Age-related dopamine D2/D3 receptor loss in extrastriatal regions of the human brain. Neurobiol. Aging 21, 683–688. 10.1016/S0197-4580(00)00149-4 11016537

[B46] Kandimalla R. Reddy P. H. (2017). Therapeutics of neurotransmitters in Alzheimer's Disease. J. Alzheimer's Dis. 57, 1049–1069. 10.3233/JAD-161118 28211810 PMC5793221

[B47] Kemppainen N. Laine M. Laakso M. P. Kaasinen V. Nagren K. Vahlberg T.. (2003). Hippocampal dopamine D2 receptors correlate with memory functions in Alzheimer's disease. Eur. J. Neurosci. 18, 149–154. 10.1046/j.1460-9568.2003.02716.x 12859348

[B48] Khoshbouei H. Sen N. Guptaroy B. Johnson L. Lund D. Gnegy M. E.. (2004). N-Terminal phosphorylation of the dopamine transporter is required for amphetamine-induced efflux. PLoS Biol. 2, 78. 10.1371/journal.pbio.0020078 15024426 PMC368172

[B49] Koch G. Di Lorenzo F. Bonn,ì S. Giacobbe V. Bozzali M. Caltagirone C.. (2014). Dopaminergic modulation of cortical plasticity in Alzheimer's disease patients. Neuropsychopharmacology 39, 2654–2661. 10.1038/npp.2014.119 24859851 PMC4207345

[B50] Koch G. Motta C. Bonn,ì S. Pellicciari M. C. Picazio S. Casula E. P.. (2020). Effect of rotigotine vs placebo on cognitive functions among patients with mild to moderate Alzheimer disease: a randomized clinical trial. JAMA Netw. Open 3, e2010372–e2010372. 10.1001/jamanetworkopen.2020.10372 32667654 PMC7364345

[B51] Koeppe R. A. Gilman S. Junck L. Wernette K. Frey K. A. (2008). Differentiating Alzheimer's disease from dementia with Lewy bodies and Parkinson's disease with (+)-[11C] dihydrotetrabenazine positron emission tomography. Alzheimer's Dement. 4, S67–S76. 10.1016/j.jalz.2007.11.016 18632004

[B52] Kouhnavardi S. Ecevitoglu A. Dragačević V. Sanna F. Arias-Sandoval E. Kalaba P.. (2022). A novel and selective dopamine transporter inhibitor, (S)-MK-26, promotes hippocampal synaptic plasticity and restores effort-related motivational dysfunctions. Biomolecules 12, 881. 10.3390/biom12070881 35883437 PMC9312958

[B53] Krashia P. Spoleti E. D'Amelio M. (2022). The VTA dopaminergic system as diagnostic and therapeutical target for Alzheimer's disease. Front. Psychiat. 13, 1039725. 10.3389/fpsyt.2022.1039725 36325523 PMC9618946

[B54] Kristofova M. Aher Y. D. Ilic M. Radoman B. Kalaba P. Dragacevic V.. (2018). A daily single dose of a novel modafinil analogue CE-123 improves memory acquisition and memory retrieval. Behav. Brain Res. 343, 83–94. 10.1016/j.bbr.2018.01.032 29410048

[B55] Kumar U. Patel S. C. (2007). Immunohistochemical localization of dopamine receptor subtypes (D1R–D5R) in Alzheimer's disease Brain. Brain Res. 1131, 187–196. 10.1016/j.brainres.2006.10.049 17182012

[B56] Kurzina N. P. Aristova I. Y. Volnova A. B. Gainetdinov R. R. (2020). Deficit in working memory and abnormal behavioral tactics in dopamine transporter knockout rats during training in the 8-arm maze. Behav. Brain Res. 390, 112642. 10.1016/j.bbr.2020.112642 32428629

[B57] Lammel S. Hetzel A. Häckel O. Jones I. Liss B. Roeper J.. (2008). Unique properties of mesoprefrontal neurons within a dual mesocorticolimbic dopamine system. Neuron 57, 760–773. 10.1016/j.neuron.2008.01.022 18341995

[B58] Lebedev A. V. Nilsson J. Lindström J. Fredborg W. Akenine U. Hillilä C.. (2020). Effects of daily L-dopa administration on learning and brain structure in older adults undergoing cognitive training: a randomised clinical trial. Scient. Rep. 10, 5227. 10.1038/s41598-020-62172-y 32251360 PMC7090037

[B59] Leo D. Sukhanov I. Zoratto F. Illiano P. Caffino L. Sanna F.. (2018). Pronounced hyperactivity, cognitive dysfunctions, and bdnf dysregulation in dopamine transporter knock-out rats. J. Neurosci. 38, 1959–1972. 10.1523/JNEUROSCI.1931-17.2018 29348190 PMC5824739

[B60] Leuner K. Schütt T. Kurz C. Eckert S. H. Schiller C. Occhipinti A.. (2012). Mitochondrion-derived reactive oxygen species lead to enhanced amyloid beta formation. Antioxid. Redox Signal. 16, 1421–1433. 10.1089/ars.2011.4173 22229260 PMC3329950

[B61] Li H. Hirano S. Furukawa S. Nakano Y. Kojima K. Ishikawa A.. (2020). The relationship between the striatal dopaminergic neuronal and cognitive function with aging. Front. Aging Neurosci. 12, 41. 10.3389/fnagi.2020.00041 32184717 PMC7058549

[B62] Loland C. J. Mereu M. Okunola O. M. Cao J. Prisinzano T. E. Mazier S.. (2012). R-modafinil (armodafinil): a unique dopamine uptake inhibitor and potential medication for psychostimulant abuse. Biol. Psychiat. 72, 405–413. 10.1016/j.biopsych.2012.03.022 22537794 PMC3413742

[B63] Madras B. K. Xie Z. Lin Z. Jassen A. Panas H. Lynch L.. (2006). Modafinil occupies dopamine and norepinephrine transporters in vivo and modulates the transporters and trace amine activity in vitro. J. Pharmacol. Exper. Therap. 319, 561–569. 10.1124/jpet.106.106583 16885432

[B64] Manczak M. Calkins M. J. Reddy P. H. (2011). Impaired mitochondrial dynamics and abnormal interaction of amyloid beta with mitochondrial protein drp1 in neurons from patients with Alzheimer's disease: implications for neuronal damage. Hum. Molec. Genet. 20, 2495–2509. 10.1093/hmg/ddr139 21459773 PMC3109997

[B65] McNamara C. G. Tejero-Cantero Á. Trouche S. Campo-Urriza N. Dupret D. (2014). Dopaminergic neurons promote hippocampal reactivation and spatial memory persistence. Nat. Neurosci. 17, 1658–1660. 10.1038/nn.3843 25326690 PMC4241115

[B66] Meyer-Luehmann M. Spires-Jones T. L. Prada C. Garcia-Alloza M. Calignon A. Rozkalne A.. (2008). Rapid appearance and local toxicity of amyloid-β plaques in a mouse model of Alzheimer's disease. Nature 451, 720–724. 10.1038/nature06616 18256671 PMC3264491

[B67] Miller D. R. Guenther D. T. Maurer A. P. Hansen C. A. Zalesky A. Khoshbouei H.. (2021). Dopamine transporter is a master regulator of dopaminergic neural network connectivity. J. Neurosci. 41, 5453–5470. 10.1523/JNEUROSCI.0223-21.2021 33980544 PMC8221606

[B68] Mishra A. Singh S. Shukla S. (2018). Physiological and functional basis of dopamine receptors and their role in neurogenesis: possible implication for Parkinson's disease. J. Exper. Neurosci. 12, 1179069518779829. 10.1177/1179069518779829 29899667 PMC5985548

[B69] Mohamad Najib N. H. Yahaya M. F. Das S. Teoh S. L. (2023). The effects of metallothionein in paraquat-induced Parkinson disease model of zebrafish. Int. J. Neurosci. 133, 822–833. 10.1080/00207454.2021.1990916 34623211

[B70] Murray A. M. Weihmueller F. B. Marshall J. F. Hurtig H. I. Gottleib G. L. Joyce J. N.. (1995). Damage to dopamine systems differs between Parkinson's disease and Alzheimer's disease with parkinsonism. Ann. Neurol. 37, 300–312. 10.1002/ana.410370306 7695230

[B71] Navaroli D. M. Stevens Z. H. Uzelac Z. Gabriel L. King M. J. Lifshitz L. M.. (2011). The plasma membrane-associated GTPase rin interacts with the dopamine transporter and is required for protein kinase c-regulated dopamine transporter trafficking. J. Neurosci. 31, 13758–13770. 10.1523/JNEUROSCI.2649-11.2011 21957239 PMC3205929

[B72] Nepal B. Das S. Reith M. E. Kortagere S. (2023). Overview of the structure and function of the dopamine transporter and its protein interactions. Front. Physiol. 14, 1150355. 10.3389/fphys.2023.1150355 36935752 PMC10020207

[B73] Nobili A. Latagliata E. C. Viscomi M. T. Cavallucci V. Cutuli D. Giacovazzo G.. (2017). Dopamine neuronal loss contributes to memory and reward dysfunction in a model of Alzheimer's disease. Nat. Commun. 8, 14727. 10.1038/ncomms14727 28367951 PMC5382255

[B74] Norrara B. Fiuza F. P. Arrais A. C. Costa I. M. Santos J. R. Engelberth R. C. G. J.. (2018). Pattern of tyrosine hydroxylase expression during aging of mesolimbic pathway of the rat. J. Chem. Neuroan. 92, 83–91. 10.1016/j.jchemneu.2018.05.004 29842891

[B75] Pahrudin Arrozi A. Wan Ngah W. Z. Mohd Yusof Y. A. Ahmad Damanhuri M. H. Makpol S. (2017). Antioxidant modulation in restoring mitochondrial function in neurodegeneration. Int. J. Neurosci. 127, 218–235. 10.1080/00207454.2016.1178261 27074540

[B76] Palop J. J. Mucke L. (2010). Amyloid-β-induced neuronal dysfunction in Alzheimer's disease: from synapses toward neural networks. Nat. Neurosci. 13, 812–818. 10.1038/nn.2583 20581818 PMC3072750

[B77] Pan X. Kaminga A. C. Wen S. W. Wu X. Acheampong K. Liu A. (2019). Dopamine and dopamine receptors in Alzheimer's disease: a systematic review and network meta-analysis. Front. Aging Neurosci. 11, 175. 10.3389/fnagi.2019.00175 31354471 PMC6637734

[B78] Perreault M. L. Hasbi A. O'Dowd B. F. George S. R. (2011). The dopamine D1–D2 receptor heteromer in striatal medium spiny neurons: evidence for a third distinct neuronal pathway in basal ganglia. Front. Neuroan. 5, 31. 10.3389/fnana.2011.00031 21747759 PMC3130461

[B79] Pizzagalli D. A. Berretta S. Wooten D. Goer F. Pilobello K. T. Kumar P.. (2019). Assessment of striatal dopamine transporter binding in individuals with major depressive disorder: in vivo positron emission tomography and postmortem evidence. JAMA Psychiatry 76, 854–861. 10.1001/jamapsychiatry.2019.0801 31042280 PMC6495358

[B80] Ramamoorthy S. Shippenberg T. S. Jayanthi L. D. (2011). Regulation of monoamine transporters: role of transporter phosphorylation. Pharmacol. Therap. 129, 220–238. 10.1016/j.pharmthera.2010.09.009 20951731 PMC3031138

[B81] Sagheddu C. Pintori N. Kalaba P. Dragačević V. Piras G. Lubec J.. (2020). Neurophysiological and neurochemical effects of the putative cognitive enhancer (S)-CE-123 on mesocorticolimbic dopamine system. Biomolecules 10, 5. 10.3390/biom10050779 32443397 PMC7277835

[B82] Sala A. Caminiti S. P. Presotto L. Pilotto A. Liguori C. Chiaravalloti A.. (2021). In vivo human molecular neuroimaging of dopaminergic vulnerability along the Alzheimer's disease phases. Alzheimer's Res. Ther. 13, 187. 10.1186/s13195-021-00925-1 34772450 PMC8588696

[B83] Salvatore M. F. Calipari E. S. Jones S. R. (2016). Regulation of tyrosine hydroxylase expression and phosphorylation in dopamine transporter-deficient mice. ACS Chem. Neurosci. 7, 941–951. 10.1021/acschemneuro.6b00064 27124386 PMC4956566

[B84] Salvatore M. F. Pruett B. S. (2012). Dichotomy of tyrosine hydroxylase and dopamine regulation between somatodendritic and terminal field areas of nigrostriatal and mesoaccumbens pathways. PLoS ONE 7, e29867. 10.1371/journal.pone.0029867 22242182 PMC3252325

[B85] Saroja S. R. Aher Y. D. Kalaba P. Aher N. Y. Zehl M. Korz V.. (2016). A novel heterocyclic compound targeting the dopamine transporter improves performance in the radial arm maze and modulates dopamine receptors D1-D3. Behav. Brain Res. 312, 127–137. 10.1016/j.bbr.2016.06.011 27288589

[B86] Schott B. H. Seidenbecher C. I. Fenker D. B. Lauer C. J. Bunzeck N. Bernstein H.. (2006). The dopaminergic midbrain participates in human episodic memory formation: evidence from genetic imaging. J. Neurosci. 26, 1407–1417. 10.1523/JNEUROSCI.3463-05.2006 16452664 PMC6675495

[B87] Selkoe D. J. (2002). Alzheimer's disease is a synaptic failure. Science 298, 789–791. 10.1126/science.1074069 12399581

[B88] Shan J. Javitch J. A. Shi L. Weinstein H. (2011). The substrate-driven transition to an inward-facing conformation in the functional mechanism of the dopamine transporter. PLoS ONE 6, e16350. 10.1371/journal.pone.0016350 21298009 PMC3029329

[B89] Shi L. Quick M. Zhao Y. Weinstein H. Javitch J. A. (2008). The mechanism of a neurotransmitter: sodium symporter—inward release of Na+ and substrate is triggered by substrate in a second binding site. Molec. Cell 30, 667–677. 10.1016/j.molcel.2008.05.008 18570870 PMC2826427

[B90] Shyu B. C. Gao Z. Y. Wu J. J. S. He A. B. H. Cheng C. N. Huang A. C. W. (2021). Methamphetamine and modulation functionality of the prelimbic cortex for developing a possible treatment of Alzheimer's disease in an animal model. Front. Aging Neurosci. 13, 751913. 10.3389/fnagi.2021.751913 34744692 PMC8564002

[B91] Sorkina T. Caltagarone J. Sorkin A. (2013). Flotillins regulate membrane mobility of the dopamine transporter but are not required for its protein kinase C dependent endocytosis. Traffic 14, 709–724. 10.1111/tra.12059 23418867 PMC3947585

[B92] Swant J. Goodwin J. S. North A. Ali A. A. Gamble-George J. Chirwa S.. (2011). α-synuclein stimulates a dopamine transporter-dependent chloride current and modulates the activity of the transporter. J. Biol. Chem. 286, 43933–43943. 10.1074/jbc.M111.241232 21990355 PMC3243541

[B93] Tarkowski E. (2003). Intrathecal inflammation precedes development of Alzheimer's disease. J. Neurol. Neurosurg. Psychiatry 74, 1200–1205. 10.1136/jnnp.74.9.1200 12933918 PMC1738668

[B94] Udo N. Hashimoto N. Toyonaga T. Isoyama T. Oyanagi Y. Narita H.. (2020). Apathy in Alzheimer's disease correlates with the dopamine transporter level in the caudate nuclei. Dement. Geriatr. Cogn. Disor. Extra 10, 86–93. 10.1159/000509278 33082772 PMC7548940

[B95] Vassilev P. Pantoja-Urban A. H. Giroux M. Nouel D. Hernandez G. Orsini T.. (2021). Unique effects of social defeat stress in adolescent male mice on the netrin-1/DCC pathway, prefrontal cortex dopamine and cognition. ENeuro 8, 45. 10.1523/ENEURO.0045-21.2021 33619036 PMC8051112

[B96] Vaughan R. A. Foster J. D. (2013). Mechanisms of dopamine transporter regulation in normal and disease states. Trends Pharmacol. Sci. 34, 489–496. 10.1016/j.tips.2013.07.005 23968642 PMC3831354

[B97] Volkow N. D. Ding Y. S. Fowler J. S. Wang G. J. Logan J.. (1996). Dopamine transporters decrease with age. J. Nucl. Med. 37, 554–559. 8691238

[B98] Wheeler D. S. Underhill S. M. Stolz D. B. Murdoch G. H. Thiels E. Romero G.. (2015). Amphetamine activates Rho GTPase signaling to mediate dopamine transporter internalization and acute behavioral effects of amphetamine. Proc. Nat. Acad. Sci. 112, E7138–E7147. 10.1073/pnas.1511670112 26553986 PMC4697400

[B99] Xiao G. Zhao M. Liu Z. Du F. Zhou B. (2021). Zinc antagonizes iron-regulation of tyrosine hydroxylase activity and dopamine production in Drosophila melanogaster. BMC Biol. 19, 1–16. 10.1186/s12915-021-01168-0 34732185 PMC8564973

[B100] Xu K. Guo J. Ge M. Yin J. Zhang H. Yin J.. (2021). Effects of dopamine transporter changes in the ventral tegmental area of the midbrain on cognitive function in aged rats. J. Chem. Neuroan. 117, 102009. 10.1016/j.jchemneu.2021.102009 34329711

[B101] Yin L. Zhou J. Li T. Wang X. Xue W. Zhang J.. (2023). Inhibition of the dopamine transporter promotes lysosome biogenesis and ameliorates Alzheimer's disease–like symptoms in mice. Alzheimer's Dementia 19, 1343–1357. 10.1002/alz.12776 36130073

[B102] Zapata A. Kivell B. Han Y. Javitch J. A. Bolan E. A. Kuraguntla D.. (2007). Regulation of dopamine transporter function and cell surface expression by D3 dopamine receptors. J. Biol. Chem. 282, 35842–35854. 10.1074/jbc.M611758200 17923483

